# Humor and Hunger Affect the Response Toward Food Cues

**DOI:** 10.3389/fpsyg.2021.680508

**Published:** 2021-09-16

**Authors:** Eva Froehlich, Larissa Samaan, Rie Matsuzaki, Soyoung Q Park

**Affiliations:** ^1^Decision Neuroscience and Nutrition, German Institute of Human Nutrition (DIfE), Nuthetal, Germany; ^2^Berlin School of Mind and Brain, Humboldt-Universität zu Berlin, Berlin, Germany; ^3^Neuroscience Research Center, Charité-Universitätsmedizin Berlin, Corporate Member of Freie Universität Berlin, Humboldt-Universität zu Berlin, and Berlin Institute of Health, Neuroscience Research Center, Berlin, Germany; ^4^Deutsches Zentrum Für Diabetes, Neuherberg, Germany

**Keywords:** attentional bias, emotional eating, food cues, humor, high caloric, hunger, low caloric, positive emotions

## Abstract

The omnipresence of food cues in everyday life has been linked to troubled eating behavior and rising rates of obesity. While extended research has been conducted on the effects of negative emotions and stress on food consumption, very little is known about how positive emotions affect eating and particularly attention toward food cues. In the present study, we investigated whether humor impacts attentional bias toward food and whether it will affect preferences for healthy and unhealthy food items, depending on the hunger state. To do so, a group of randomly assigned participants watched funny video clips (humor group, *N* = 46) or neutral ones (control group, *N* = 49). Afterwards, they performed a modified Posner cueing task with low or high caloric food images serving as cues. We found a significant group × hunger interaction. Compared to the control group, the humor group responded more slowly to food cues when hungry, whereas the opposite was true when participants were satiated. Additionally, our results suggest that hunger possibly directs attention away from healthy food cues and toward unhealthy ones. No group differences were found with respect to food preferences and engagement and disengagement of attention. We discuss the potential of humor in counteracting aversive consequences of hunger on attention allocation toward food. We propose an underlying mechanism involving a combined reduction in cortisol levels and a decrease in activation of the reward system. However, given the novelty of the findings, further research is warranted, both to replicate the results as well as to investigate the suggested underlying processes.

## Introduction

Previous studies suggest multiple potential mechanisms of humor and laughter benefiting health (e.g., Martin, 2002). Humor attenuates the adverse effects of stress (Martin, 2002). Furthermore, it has been hypothesized that laughter produces beneficial physiological changes and that positive mood induced by humor enhances the immune system (Barak, 2006; Hasan and Hasan, 2009) and dampens cardiovascular consequences of negative emotions (Fredrickson et al., 2000). However, while extensive research has been conducted on the effects of adverse emotions and sensations (such as stress) on eating and drinking (e.g., Macht, 2008; Cardi et al., 2015; Evers et al., 2018; Reichenberger et al., 2020), considerably less is known about the influence of humor and the resulting positive mood on eating (Evers et al., 2013; Bast and Berry, 2014). Given the benefits of a healthy diet for physical and mental health (AlAmmar et al., 2020), this research gap seems rather surprising. The aim of the current study is to investigate how humor affects attention toward healthy and unhealthy food cues.

Every day, we are confronted with numerous advertisements for foods, drinks and snacks. Often, these ads promote unhealthy, highly processed foods (Harris et al., 2009a). This plethora of highly palatable food cues in today's environment is thought to be responsible for the rise in obesity rates and for problems in regulating eating behavior (Harris et al., 2009b; Meule and Vögele, 2013; Blechert et al., 2014a). One possible contributing factor in this relationship is the attentional bias toward food (Jonker et al., 2020). It has been suggested that the highly rewarding value of food (e.g., Petrovich et al., 2005; Brown and Park, 2020) captures one's attention automatically, heightens response activation, and increases approach behavior (Higgs et al., 2017; Jonker et al., 2020). For healthy-weight individuals, food deprivation increases the reward value attributed to food, while satiation decreases it (Higgs et al., 2017). Accordingly, healthy-weight individuals show an attentional bias toward food cues only when they had fasted yet not when being sated (Castellanos et al., 2009). Similarly, the attentional bias of healthy-weight individuals with higher self-reported hunger levels was found to be greater than that of those with lower hunger levels (Mogg et al., 1998).

A bias for attentional engagement (i.e., attentional bias *toward* food cues), has been linked to healthy eating behavior as demonstrated by Jonker et al. (2019). In their study, patients with Anorexia Nervosa lacked an attentional engagement bias for briefly presented food cues when compared to healthy eating adolescents, yet they showed no difference in attentional disengagement. Distinct from attentional engagement, attentional disengagement describes the process of preparing to direct attention *away* from food cues (e.g., Posner and Petersen, 1990; Tapper et al., 2010; Jonker et al., 2019). While Jonker et al. (2020) reported no differences in attentional disengagement between fasted and satiated individuals, Tapper et al. (2010) showed that hunger as well as reward-drive were predictive of delayed attentional disengagement for shortly presented food cues. Independent of these somewhat inconsistent findings, attentional disengagement has been considered an important factor in eating behavior, capturing mechanisms that can be differentiated from those underlying attentional engagement (Jonker et al., 2020), and thus calling for a systematic investigation of both processes when studying attentional bias toward food cues.

Besides attentional engagement and disengagement, the affective state of individuals is another important factor to be taken into account, due to its potential influence on attention allocation. For instance, Wadlinger and Isaacowitz (2006) demonstrated that, compared to neutral mood, participants experiencing positive mood attended to peripheral images more frequently. Individuals experiencing negative emotions, such as anxiety, are more likely to attend to negative or threatening stimuli, rather than positive (Mogg et al., 2000). Hepworth et al. (2010) investigated the impact of negative affect on attentional bias toward food cues and showed that negative emotions increase attention allocation toward food cues as well subjective appetite (i.e., self-reported hunger or the urge to eat) compared to a neutral mood state. Based on the positive correlation between attentional bias and subjective appetite, they propose a common underlying mechanism. Yet, to the best of our knowledge, no research has been so far conducted on the effect of emotions on attentional disengagement and the influence of positive emotions on attention allocation toward food cues in general.

Extensive research, however, has been conducted on the relationship between emotional states and food intake, with the bulk of research focusing on the impact of negative emotions (i.e., emotional eating; e.g., Reichenberger et al., 2020). Remarkably, only recently has research started to recognize the role of positive emotions (e.g., Bongers et al., 2013), although findings on that topic are, so far, somewhat inconsistent. Compared to a neutral mood, positive mood has been shown to increase intake of unhealthy foods and sweet drinks (Evers et al., 2013; Winkielman et al., 2015) and lead to increased eating behavior in general (Cardi et al., 2015; Evers et al., 2018). On the other hand, positive mood has been suggested to attenuate hunger stimuli (Bast and Berry, 2014) and accordingly, positive emotions may attenuate food intake in individuals with a more controlled eating behavior (Turner et al., 2010), or have no effect on it at all (Lowe and Fisher, 1983; Yeomans and Coughlan, 2009). Further, social eating, which is often accompanied by positive mood, can promote either under- or overeating, depending on the context (Young et al., 2009; Howland et al., 2012).

In general, most studies that investigate the influence of positive emotions on eating behavior focus on the *amount* or *frequency* of food intake and when it comes to *preferences* made between different types of food on savory and/or sweet foods (e.g., popcorn, sandwiches, chips, cookies, chocolate or ice-cream; Cardi et al., 2015; Evers et al., 2018). One of the few previous studies which experimentally investigated the impact of positive mood on ratings of healthy and unhealthy food items, found that individuals in a positive mood assigned higher ratings to healthy food items (Gardner et al., 2014). However, no such distinction was reported for the preference between chocolate and coconut water (Andrade, 2005).

In summary, an attentional engagement bias toward food when one is hungry seems to be a part of healthy eating behavior. Negative mood has been found to affect attention allocation toward food cues. It has yet to be shown how positive emotions influences both attentional engagement and disengagement toward food items and how hunger factors into this equation. Furthermore, very little is known about the effect of positive affect on the preference for healthy and unhealthy food items. Therefore, the aim of the present study was to (a) investigate whether humor and thus positive emotions affect attention allocated toward food, (b) whether there are differences in attentional engagement and disengagement as a function of humor, and (c) whether humor affects preferences for healthy and unhealthy food.

For this purpose, we randomly assigned participants to a humor or a control group. Mood was induced using funny or neutral video clips and monitored *via* mood assessments before and after watching the clips. Allocation of attention toward food cues was measured using a modified Posner cueing task (cf. Posner, 1980). Food images depicting either high caloric or low caloric food from the Salzburg Food Pics database (Blechert et al., 2014a) served as cues. Food pictures and cues have been proven to be highly efficient for researching underlying mechanisms of food intake (Blechert et al., 2014b; Meule et al., 2014), and have been shown to be comparable to real food in predicting eating and weight gain (Blechert et al., 2019). The modified Posner task (food cue attention task; FCAT) was chosen because it allows both the assessment of attentional engagement toward food cues as well as disengagement away from food cues. Following the FCAT, participants completed questionnaires relating to health status, trait humor, and eating behavior to control for possible confounds. Additionally, we obtained hunger ratings and the hours since the last meal, as they have been shown to potentially impact attention toward food cues. Due to the Covid-19 pandemic, the study was conducted online.

As negative emotions have been shown to affect attentional bias toward food, our primary hypothesis was that this may also hold true for positive emotions. We expected humor to affect attention allocated toward food, indicated by group differences in response times for in the FCAT. Further, if attentional engagement differs from disengagement as a function of group, we assume a significant interaction of these two factors. In addition, according to the broaden-and-build theory (Fredrickson, 1998, 2001), positive emotions widen the thought-action repertoire, and expand the scope of attention, whereas negative emotions narrow it. This mindset may make it easier to resist highly palatable food (Evers et al., 2013) and may lead individuals to consider a broader range of foods, including healthy food options. If humor has an effect on food choice, there would be a significant interaction of group and food cues. Finally, a significant three-way interaction would indicate that humor affects attention orientation toward or away from healthy food differently than that of unhealthy food cues. We deliberately refrained from specifying the direction of the expected effects as previous research and theories predict conflicting outcomes. In line with the broaden-and-build theory (Fredrickson, 1998, 2001), we would expect a broadening of attention and an increase in personal resources, which would lead to faster response times in the humor group. In contrast, while humor captures people's attention (Madden and Weinberger, 1982; Koneska et al., 2017) and reduces negative cognition (Djambaska et al., 2016), it can also effectively distract attention (vampire effect; e.g., Weinberger et al., 1995), possibly resulting in an unspecific allocation of attention toward food cues, which may then lead to a disadvantage for the humor group.

## Method

### Participants

A total of 112 participants initially completed the study. Half of them were randomly assigned to the humor group (*N* = 56,32.1% female), and half of them to the control group (*N* = 56,32.1% female). Participants were required to meet the following customized prescreening criteria set on Prolific: between 18 and 50 years of age, German as first language, no current or prior mental illnesses, no dietary restrictions and being a healthy weight (body mass index [BMI] = 18–24.9 kg/m^2^). As a consequence of the ongoing Covid-19 lockdown within Germany, we decided to reassess mental health status using the short form of the German version of the PRIME MD Brief Patient Health Questionnaire (Kurzform PHQ-9; Kroenke et al., 2001; Löwe et al., 2002), which is a screening tool for symptoms of major depression and depressive disorders. We also calculated participants' BMI based on their weight and height. Following this assessment, we had to exclude 17 participants as they were showing medium to severe symptoms of major depression (scores between 10 and 20). Of the final sample of 95 participants, 46 were in the humor group (26.1% female; *M*_age_ = 27.1, range 18–40; *M*_BMI_ = 22.7 kg/m^2^, range 19.5–28.9) and 49 were in the control group (34.7% female; *M*_age_ = 26.6, range 18–44; *M*_BMI_ = 22.1 kg/m^2^, range 18.7–26.1). Differences in distribution of sex and means/medians of age and BMI were non-significant across groups. Participants were screened and recruited *via* the research platform Prolific (www.prolific.co) and tested *via* pavlovia (pavlovia.org). The study took place between January 21 and January 29, 2021. Prior to completion of the study, participants gave their informed consent, and after completion they received £6.72 (~7.50 Euro) as compensation. The study was conducted in accordance with the Code of Ethics of the World Medical Association and approved by the ethics committee of the Humboldt-Universität zu Berlin.

### Stimuli and Material

#### Mood Assessment

Mood assessment took place twice, at the beginning of the study and after watching the video clips. Participants were asked to rate their current mood using the Positive and Negative Affect Scale (PANAS; Janke and Glöckner-Rist, 2014). The PANAS contains 20 items, 10 of which describe positive emotions and feelings (e.g., “proud”) and 10 negative ones (e.g., “scared”). Answers are given on a 5-point Likert scale (1 = “not at all,” 5 = “very much”) with higher scores indicating higher positive or negative affect. The sums of the positive and negative item scores served as dependent variables in the present study.

#### Homeostatic State

As hunger has been found to reliably influence the attention given to food cues (e.g., Castellanos et al., 2009; Tapper et al., 2010; Jonker et al., 2020), participants reported their current state of hunger as well as thirst on a visual analog scale (VAS) anchored by 0 (not hungry at all) and 100 (extremely hungry) right after mood assessment. Additionally, they specified the time that had passed since their last meal. Higher VAS values imply higher hunger and thirst levels, higher time values denote longer fasting periods. Hunger ratings were used as a predictor variable in the present study (cf. data analysis section).

#### Mood Induction

Depending on the condition, participants saw either a sequence of humorous or neutral video clips. Both sequences had a total duration of ~9 min 30 s. To ensure participants actively watched the clips, after every 3 min they had to press a button for the videos to continue. The time participants spent watching the video clips was recorded and checked for plausibility. Humorous clips depicted small, harmless accidents or mishaps of toddlers, children and adolescents, or animals behaving oddly. In the control condition, participants watched excerpts of documentaries covering different topics, such as extinct animals, or building an oven. The video clips have been used in our lab before with humorous clips being rated funnier than neutral ones (Froehlich et al., 2021).

#### Video, Valence, and Arousal Assessment

To ensure the effectiveness of the video clips, participants rated the clips' funniness on a 10-point scale (1 = not funny, 10 = very funny) after watching them. Additionally, we asked on a 4-point scale whether participants showed a physiological response while watching the clips (1 = “I did neither smile nor laugh,” 2 = “I had to smile,” 3 = “I had to chuckle,” 4 = “I had to laugh out loud”). As the level of arousal may interact with or even drive the proposed effect of humor by means of distraction (e.g., Martin, 2002), participants additionally indicated their level of arousal and valence as well as that of the video clips on a 7-point Likert scale using Self-Assessment Manikins (SAMs; Bradley and Lang, 1994). Higher scores indicate a higher level of arousal or valence. The scores of each of the scales served as dependent variables.

#### Food Cue Attention Task

A total of 88 images from the Salzburg food pic database (Blechert, Blechert et al., 2014b; Meule et al., 2014) were chosen for this study. Of those, 44 images showed low caloric food items (*M* = 55.6 kcal/100 g, range 9–123), thus representing healthy food options, whereas 44 images showed high caloric foods (*M* = 320.3 kcal/100 g, range 139–654), intended to represent unhealthy food options. The difference in caloric content between low and high caloric food images was highly significant, *t*_(48.9)_ = 12.2, *p* < 0.001, *r* = 0.868. The images of both categories showed processed and whole food items as well as sweet and savory food items. 68.2% of the food stimuli in the low caloric condition depicted whole foods, compared to 6.8% in the high caloric condition. Using Fisher's exact test this difference in the processing state of food was found to be significant, *p* < 0.001. The difference in taste was found to be non-significant as indicated by Pearson's chi-squared test, χ^2^(2) = 2.27, *p* = 0.32 with 50.0% of the low and 45.5% of the high caloric images showing savory stimuli. For 20.5% of the images, no data regarding taste were acquired. More importantly, we carefully matched high and low caloric food stimuli with respect to physical image properties such as contrast, complexity, size, color, and intensity as well as for food image properties such as total caloric content, valence, arousal, craving, and familiarity, all *p*s > 0.08. Items and item characteristics are listed in the supplementary material (Supplementary Tables 1, 2).

#### Eating Behavior Questionnaires

To control for possible group differences in emotional and stress eating behaviors, we administered the Salzburg Emotional Eating Scale (SEES; Meule et al., 2018a) as well as the Salzburg Stress Eating Scale (SSES; Meule et al., 2018b). Moreover, as subjective appetite has been found to affect attention toward food cues (at least when being in a negative mood; Hepworth et al., 2010), participants additionally filled out the Food Craving Questionnaire State (FCQ-S; Meule et al., 2014). The SEES consists of 20 items, separated into four subscales (5 items each) measuring emotional eating due to happiness, sadness, anger, and anxiety. Participants indicate on a 5-point scale whether they eat less, the same, or more, when feeling certain emotions (1 = I eat significantly less, 5 = I eat significantly more). Mean scores above 3 indicate increased emotional eating, mean scores around 3 indicate equivalent eating, and mean scores below 3 indicate eating less when feeling the specific emotion. The total mean score as well as the mean scores for the subscales served as dependent variables. The SSES consists of 10 items for which participants rate their changes in eating behavior as a response to various stressful situations on a 5-point scale (1 = I eat significantly less, 5 = I eat significantly more). Identical to emotional eating, mean scores above 3 indicate an increase in eating when being stressed, mean scores around 3 indicate eating as usual and mean scores below 3 indicate a decrease in food intake. In the present study mean scores were used to compare groups. The FCQ-S assesses the intensity of situational food craving. Participants are asked to specify on a 5-point scale how strongly they agree (5) or disagree (1) with 15 different statements (e.g., “When I eat something now, my stomach wouldn't feel as empty.”). Higher scores indicate higher craving. Mean scores were used as the dependent variable.

#### Health and Personality Questionnaires

The present online study took place during the second lockdown in Germany during the Covid-19 pandemic. Given the severe consequences for mental health and physically well-being (e.g., Bäuerle et al., 2020; Fiorillo et al., 2020; Jung et al., 2020), we aimed to control for possible confounding factors affecting mood and eating behavior. For this reason, feeling of loneliness, severity of depressive symptoms as well as somatic complaints (e.g., sleep disturbance, pain, constipation, digestive complaints) were assessed using the UCLA loneliness scale (Döring and Bortz, 1993) as well as the PHQ-9 (depression) and the PHQ-15 (somatic symptoms; Kroenke et al., 2002; Löwe et al., 2002). The UCLA loneliness scale consists of 20 items and measures feelings of loneliness on a 5-point scale (1 = not at all, 5 = absolutely). The higher the score, the higher the perceived loneliness. Mean scores were used as dependent variables. The PHQs consist of nine and 15 items, respectively. The PHQ-9 yields a 4-point scale, on which participants indicate how often they experience a range of depressive symptoms (0 = not at all, 3 = almost every day). Scores below 5 indicate the absence of any depressive symptoms, scores between 5 and 10 indicate mild depressive symptoms and scores above 10 point toward the presence of a major depression of varying severity. The PHQ-15 yields a 3-point scale, on which participants specify how much they are affected by various somatic symptoms (0 = not affected, 2 = strongly affected). Higher scores indicate higher somatic symptomatology. The sum of scores served as the dependent variable for both questionnaires.

As reward drive (measured *via* the Behavioral Activation Scale drive subscale) has been shown to correlate with attentional bias scores (Tapper et al., 2010), participants filled out the Behavioral Inhibition System (BIS)/Behavioral Approach System (BAS) scales (Strobel et al., 2001). Contrary to the English version, the German BIS/BAS scales yield a two-factor structure (BIS and BAS). The BIS scale consists of seven items, the BAS scale 13 items, four items are dummy items. The items are scored on a 4-point scale (1 = does not apply to me at all, 4 = exactly applies to me). The higher the score on the BIS scale, the higher the tendency to avoid unpleasant outcomes, and the higher the score on the BAS scale, the higher the tendency to approach favorable outcomes. Mean scores of each scale were used for analyses.

Finally, to ensure, that participants of the humor and control group did not differ with respect to trait humor, we administered the Humor Styles Questionnaire (Ruch and Heintz, 2016). The questionnaire contains 32 items, which measure four different styles of humor (affiliative, aggressive, self-defeating, or self-enhancing). Items are rated on a 7-point Likert scale (1 = totally disagree, 7 = totally agree). A higher score indicates a higher usage of this particular humor style. Mean scores per subscale served as dependent variables.

### Procedure

**Figure 1:**
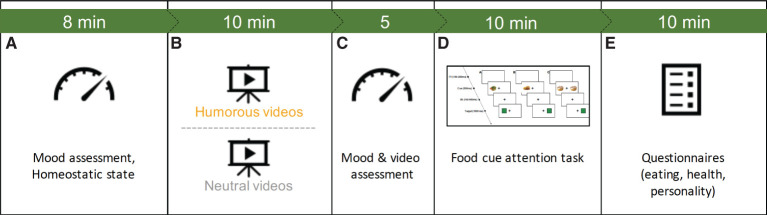
Study procedure showing the five phases of the experiment: A, initial mood and homeostatic state assessment; B, mood induction *via* video clips; C, (re-)assessment of mood, self and videoclips, D, food cue attention task and E, self-assessments *via* questionnaires. The green bar denotes the approximated durations of the phases in minutes.

The online study involved five phases: (a) initial mood and homeostatic state assessment, (b) mood induction *via* video clips, (c) (re-)assessment of mood, self and videoclips, (d) food attention task, and (e) self-assessments *via* questionnaires (Figure 1).

To keep viewing and response conditions comparable, all participants were asked to keep a viewing distance of 60 cm and usage of tablets and smart phones was technically disabled, so that only laptop or desktop computers could be used.

After giving consent and specifying their initial mood (PANAS) and homeostatic state, participants watched either the humorous video clips (humor group) or the neutral ones (control group). After again filling out the PANAS, participants additionally scored funniness, arousal and valence of the video clips as well rated their individual state of valence, arousal and whether they laughed or not.

**Figure 2:**
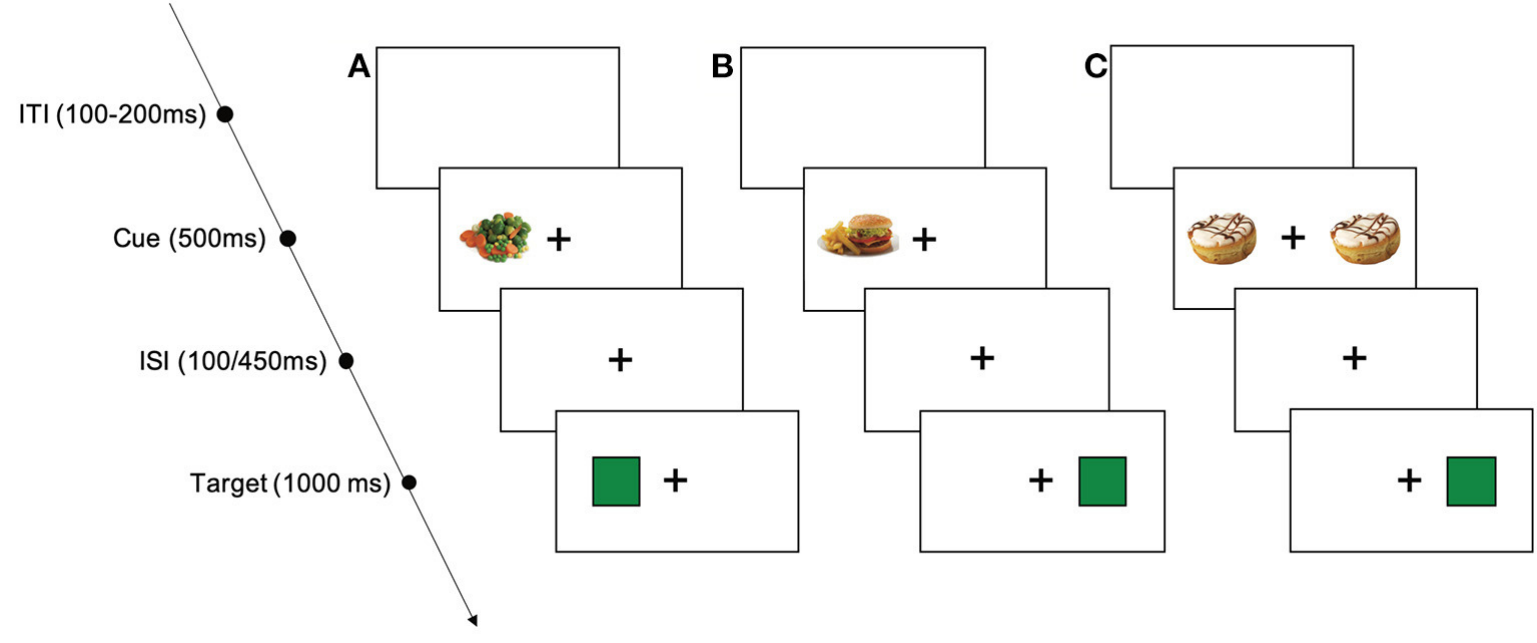
Schematic procedure of the food cue attention task (FCAT): **(A)**, example of a valid trial with a low caloric food cue; **(B)**, example of an invalid trial with a high caloric food cue; **(C)**, example of a catch trial with high caloric food cues. ITI, intertrial interval; ISI, interstimulus interval.

To assess whether humor impacts the attention allocated toward food cues, we used a modified Posner cueing paradigm (see Figure 2), the FCAT. In the FCAT, participants were asked to fixate on a cross presented centrally on the computer screen. For 500 ms, a cue depicting either a high or a low caloric food item appeared on either the left or the right side of the fixation cross. After an interstimulus interval of 100 or 450 ms, a target in the form of a green square appeared either at the same side as the cue (valid) or on the opposite side of the cue (invalid). Participants were asked to respond as quickly and as accurately as possible when the target was presented by pressing either the left or the right “arrow” key on their keyboard. The next trial started with the button press or after 2000 ms in case no response was given. An intertrial interval of 100–200 ms was used. In total, participants saw 176 trials involving 44 low and 44 high caloric food cues which were each presented twice over the course of the task. The 88 trials per food cue category were further split up into 40 trials with valid food cues, 40 trials with invalid food cues and 8 catch trials. During catch trials, cues appeared on both sides of the fixation cross and participants had to inhibit their response. The order of trials and the order of food cues were randomized for all participants. Accuracy rates and response times were used as dependent variables for further analyses. Before the actual task, participants underwent 12 training trials with feedback.

We employed exogeneous cues as we were interested in the initial bottom-up response toward food cues (e.g., Chica et al., 2014). However, to make sure participants were able to consciously perceive food cues and thus the depicted items, we refrained from using very short presentation times (<500 ms) for the food cues. Catch trials and the two randomly interspersed interstimulus intervals were used to prevent participants from rhythmically responding (i.e., from giving anticipated rather than real responses).

The food attention task was chosen as it enables us to not only assess attention toward food cues as a function of humor (differences in response times/accuracy times between groups), but because it also allows us to determine whether humor affects the (automatic) attention allocated to food items and therefore food choice (differences in response times/accuracy rates to high and low caloric food cues as a function of group). Lastly, by evaluating response times/accuracy rates for validly vs. invalidly cued high and low caloric food cues as a function of group, we are able to investigate whether humor affects initial attentional engagement toward low and high caloric food items and/or the attentional disengagement away from these food cues.

After the food attention task, participants filled out the eating behavior, health and personality questionnaires and gave demographic information regarding their age, sex, weight, and height. The order of the questionnaires was randomized across participants.

The entire study was programmed using PsychoPy3, release 2020.2.5 (Peirce et al., 2019). Questionnaires were incorporated into the study with the help of the form.io form builder (https://formio.github.io/formio.js/app/builder) and the code provided by Thomas Pronk (https://gitlab.pavlovia.org/tpronk/demo_embed_html). The code for the randomization of food stimuli and conditions in the food attention task was based on an online tutorial by Jazon Obzuko (https://youtu.be/toQ2enxAv1E).

### Data Analysis

#### Initial Quality Check of Data

As an initial quality check, we monitored the completion time of the study. The average completion time was roughly 45 min. Three participants who took more than 90 min were individually asked for reasons for the delay, after which one of the participants was excluded (prolonged break between watching the video clips and doing the cognitive food task). Eight participants finished the study in 35 min or less (*N*_humor_ = 4; *N*_control_ = 4). Here, we checked the number of erroneous responses in the food cognition task and assessed visually the response pattern for the questionnaire data. As no apparent anomalies were detected, these subjects were included in all further analyses.

#### Questionnaire and Rating Data

Before each test, we tested for normal distribution of the data within each group. When the data of both groups were normally distributed, we chose parametric tests for independent samples. When the data of both groups were non-normally distributed, we chose nonparametric tests. If the data of one of the groups were normally distributed, yet the other weren't, we followed the rule of thumb to use nonparametric tests for ordinal scaled data (i.e., scores) and parametric tests for interval scaled data (i.e., means). Therefore, we conducted nonparametric tests for independent samples (Wilcoxon rank-sum tests) to analyze scores of questionnaire data and *t*-tests for independent samples when we compared respective means. To investigate whether we successfully induced positive mood in the humor group after the presentation of the video clips, a mixed 2 × 2 ANOVA with the between subject factor group (humor vs. control) and the within subject factor time (before and after mood induction) was calculated for initial and second PANAS scores.

#### Analyses of Response Times and Accuracy

Prior to analyzing response time and accuracy data, we classified response times faster than 100 ms as anticipatory and therefore as incorrect responses (9 data points). We further planned to exclude subjects that gave more than 40% of erroneous responses, however, given a maximum error rate of 21.6%, all subjects remained in the data set. We chose to include those subjects that were not able to inhibit responses to the majority of catch trials (*N*_humor_ = 2; *N*_control_ = 6), as they did not show any higher erroneous responses to the valid/invalid trials than other participants (valid trials: 0–13.75%; invalid trials: 0–23.8%). Evidently, these subjects performed properly on those trials, that were relevant for the major analysis, i.e., the effect of hunger and mood on response times toward food cues. Finally, we excluded response times 3 *SD*s outside of the individual mean of each food cue × validity experimental cell (1.53%; cf. Hepworth et al., 2010; Vermeer et al., 2020 for exclusion criteria).

Response time data were analyzed using a linear mixed-effect model (e.g., Baayen et al., 2008). Linear mixed-effect models have been intensely in use in various publications instead of the traditional ANOVA approach in the last decades (cf. Baayen et al., 2008; Bell et al., 2019; Schad et al., 2020), and have been applied (amongst others) to assess attentional cueing effects (e.g., Kliegl et al., 2011; Chauhan et al., 2017; MacInnes and Bhatnagar, 2018; Malevich et al., 2018). One of the big advantages of linear mixed models is that one can make use of the full data set, i.e., one does not lose information due to averaging, they allow for the estimation of random effects, missing data points cause no problem, and we can model random slopes, i.e., different effects for each level of the grouping variable.

In the present study, group (humor vs. control), food cue (high vs. low caloric), validity (valid vs. invalid) and hunger ratings served as fixed effects, participants as random effects and validity as random slope. Main effects and interactions of fixed effects were tested for significance using type III Wald chi-squared tests. The final model was identified in a two-step procedure. First, we tested which factors would significantly improve a minimal linear mixed model consisting of the fixed factors group, food cue and validity as well as the random effect participants. We then added all combinations of the factors hunger ratings and self-arousal. Hunger ratings were incorporated to account for the fact that satiated normal weight individuals no longer demonstrate an attentional bias for food (e.g., Castellanos et al., 2009; Jonker et al., 2020), whereas self-arousal ratings were considered, as it is thought to accompany mood induction (Martin, 2002) and has been discussed as an influence on emotional eating (e.g., Macht, 2008). Chi-squared tests and the AIC/BIC criteria were used to identify the best fitting model. In the second step, we then added random slopes of the variables stepwise to the model and determined the best fitting model structure as in step one.

Accuracy data were analyzed using a logistic mixed effect model with group (humor vs. control), food cue (high vs. low caloric), validity (valid vs. invalid) and hunger ratings as fixed effects, participants as random effects and validity as random slope. Main effects and interactions were assessed using type III Wald chi-squared tests and z-approximation. To determine the best fitting model structure, we proceeded with the identical steps taken for response time data.

All analyses were run in R-Studio (RStudio Team, 2020). To analyze the mixed-effects models, we used the lme4 package by Bates et al. (2014). Interactions and contrasts were additionally assessed using the emtrends function from the emmeans package (Lenth, 2020). ANOVAs were calculated by means of the afex package (Singmann et al., 2020).

## Results

### Questionnaire Data and Homeostatic State

We found no group differences with respect to eating behavior in terms of stress eating (SSES) and food craving (FCQ-S). An impact of group on emotional eating (SEES) per se was observed, *t*_(90.9)_ = 2.39, *p* > 0.05, *r* = 0.244, with the humor group scoring lower on overall emotional eating than the control group. Additional difference testing including the four subscales of the SEES revealed a marginally significant effect for anxiety, *t*_(89.5)_ = 2.25, *p* = 0.05, *r* = 0.231, after correcting for multiple testing. As before, the humor group scored lower in comparison to the control group. No other subscales were found to be significantly affected by group, all *p*s > 0.37. Moreover, both groups showed no differences regarding health status, i.e., with respect to loneliness (UCLA loneliness scale), to depression (PHQ-9) and to somatic symptoms (PHQ-15). Further, there were no group differences in the motivation to approach appetitive outcomes or to avoid aversive outcomes (BIS/BAS scales). Most importantly, the groups did not differ in sense of humor (HSQ32) and their homeostatic state (hunger and thirst ratings), all *p*s > 0.10. All reported results and group means are shown in Tables 1–3.

**Table 1:** Group characteristics with respect to homeostatic state and eating behavior.

	**Control group**		**Humor group**			
	***M (SD)***	**Range**	***M (SD)***	**Range**	***t***	***p***
*Physiological data*						
Hunger	30.93 (3.65)	1–77.0	31.12 (3.40)	1–78.4	−0.04	0.94
Thirst	31.84 (3.39)	1–77.1	28.16 (3.25)	1–83.6	0.78	0.44
Time last meal (hrs)	4.07 (0.62)	0.5–16.1	4.87 (0.77)	0–15	−0.81	0.42
*Emotional Eating (SEES)*						
Total score	2.90 (0.05)	2.05–3.8	2.75 (0.04)	2.15–3.3	2.39	0.04^a^
Happiness	3.14 (0.05)	2.4–4.6	3.10 (0.07)	2–4	0.57	1.00^a^
Sadness	3.24 (0.10)	2–4.6	3.08 (0.10)	1.8–4.06	1.01	0.55^a^
Anger	2.64 (0.07)	1.6–3.8	2.50 (0.08)	1.2–3.8	1.33	0.38^a^
Anxiety	2.59 (0.09)	1–4	2.32 (0.07)	1.2–3.6	2.25	0.05^a^
*Stress Eating (SSES)*						
Total score	2.81 (0.08)	1.5–4.3	2.61 (0.09)	1.3–4.0	1.65	0.10
*Food Craving (FCQ-S)*						
Total score	2.19 (0.12)	1–4.07	2.14 (0.12)	1–3.8	0.29	1.00^a^
Desire/Lack of control	2.13 (0.12)	1–4	2.00 (0.13)	1–4	0.71	0.95^a^
Reinforcement	2.22 (0.13)	1–4.33	2.25 (0.12)	1–3.83	−0.16	1.00^a^
Hunger	2.27 (0.17)	1–5	2.22 (0.15)	1–4.33	0.21	1.00^a^

*M, mean; SD, standard deviation; t, t-test for independent samples; SEES, Salzburg Emotional Eating Scale (Meule et al., 2018a); SSES, Salzburg Stress Eating Scale (Meule et al., 2018b); FCQ, Food Craving Questionnaire—State (Meule et al., 2014)*.

a *p-value adjusted for multiple testing (Holm's correction)*.

**Table 2:** Group characteristics with respect to physical and mental health.

	**Control group**		**Humor group**			
	***M (SD)***	**Range**	***M (SD)***	**Range**	***t***	***p***
*UCLA Loneliness scale*						
Total score	2.07 (0.10)	1.25–4.2	2.13 (0.09)	1.15–3.45	−0.48	1.00^a^
Loneliness	2.00 (0.11)	1–4.67	2.00 (0.10)	1–3.67	0.03	1.00^a^
Emotional isolation	1.87 (0.11)	1–4.4	1.92 (0.10)	1–3.8	−0.32	1.00^a^
Social isolation	2.33 (0.10)	1.17–4.5	2.51 (0.10)	1.33–4	−1.30	0.39^a^
*Depressive symptoms (PHQ-9)*						
Score	5.86 (0.48)	0–15^b^	6.72 (0.49)	0–14	−1.25	0.21
*Somatoform symptoms (PHQ-15)*
Score	0.82 (0.20)	0.00–7.00	0.54 (0.16)	0.00–4.00	1.06	0.29

*M, mean; SD, standard deviation; t, t-test for independent samples. PHQ-9, Patient Health Questionnaire (depression; Kroenke et al., 2001; Löwe et al., 2002). PHQ-15, Patient Health Questionnaire (somatic symptoms; Kroenke et al., 2002; Löwe et al., 2002)*.

a *p-values adjusted for multiple testing (Holm's correction)*.

b *Participants who scored 2 or higher on the first two items and had a total score > 10 were excluded. All other participants with a total score > 10 were included*.

**Table 3:** Group characteristics with respect to approach and avoidance behavior as well as trait humor.

	**Control group**		**Humor group**			
	***M (SD)***	**Range**	***M (SD)***	**Range**	***t***	***p***
*BIS/BAS scales*						
BIS scale	2.81 (0.07)	1.57–3.86	2.97 (0.09)	1.43–4	−1.40	0.16
BAS scale	3.02 (0.05)	2.23–3.85	3.05 (0.04)	2.46–3.77	−0.36	0.72
*Sense of humor (HSQ32)*						
Total score	4.24 (0.08)	3.06–5.31	4.19 (0.09)	2.69–5.22	0.43	1.00^b^
Affiliative	5.55 (0.12)	4–6.88	5.46 (0.11)	4–7	0.53	1.00^b^
Self-enhancing	4.44 (0.14)	1.88–7	4.60 (0.15)	2.25–7	−0.74	0.92^b^
Aggressive	3.55 (0.13)	1.75–5.62	3.45 (0.13)	1.62–5.5	0.53	1.00^b^
Self-defeating	3.43 (0.14)	1.12–5.25	3.24 (0.16)	1–5.12	0.89	0.76^b^

*M, mean; SD, standard deviation; t, t-test for independent samples. BIS/BAS Scales, Behavioral Inhibition System/Behavioral Approach System (Strobel et al., 2001). HSQ32, Humor Styles Questionnaire (Ruch and Heintz, 2016)*.

a *p-value adjusted for multiple testing (Holm's correction)*.

To sum up, with the exception of emotional eating, participants do not differ in potentially confounding variables regarding homeostatic state, trait humor, eating behavior, health status and approach-avoidance behavior. Participants in the humor group showed a lesser tendency of emotional (over) eating than participants in the control group. Particularly, eating out of anxiety seems to be different for the two groups, again with the humor group eating less than the control group. Most interestingly, while the mean scores of the control and humor groups indicate that participants tend to under eat out of anxiety (*M* < 3), for overall emotional eating this holds true only for the humor group, whereas participants of the control group seem to eat the same amount as usual (*M* ~ 3).

### Mood Manipulation Check

To assess whether the video clips shown in the humor condition were indeed perceived to be funnier than those shown in the control condition, we first compared funniness and laughter ratings from the humor group to those of the control group. Results showed a significant difference between funniness ratings, *W* = 239.5, *p* < 0.001, *r* = −0.694, and laughter ratings, *W* = 296, *p* < 0.001, *r* = −0.683, between both groups. Participants from the humor group rated the clips funnier than those of the control group. Additionally, they indicated that they laughed more intensely than the control group while watching the clips (see also Table 4).

**Table 4:** Group characteristics with respect to mood manipulation.

	**Control group**		**Humor group**			
	***Med***	**Range**	***Med***	**Range**	***W***	***p***
Funniness ratings	3	1–8	7	3–10	239.5	<0.001
Laughter ratings	1	1–4	3	2–4	296	<0.001
Video valence ratings	6	4–7	5.5	3–7	1108	0.44
Self valence ratings	5	2–7	5.5	3–7	909	<0.05
Video arousal ratings	3	1–7	5	2–7	637	<0.001
Self arousal ratings	3	1–6	4	1–7	603	<0.001

*Med, median; W, Wilcoxon rank-sum test*.

**Figure 3:**
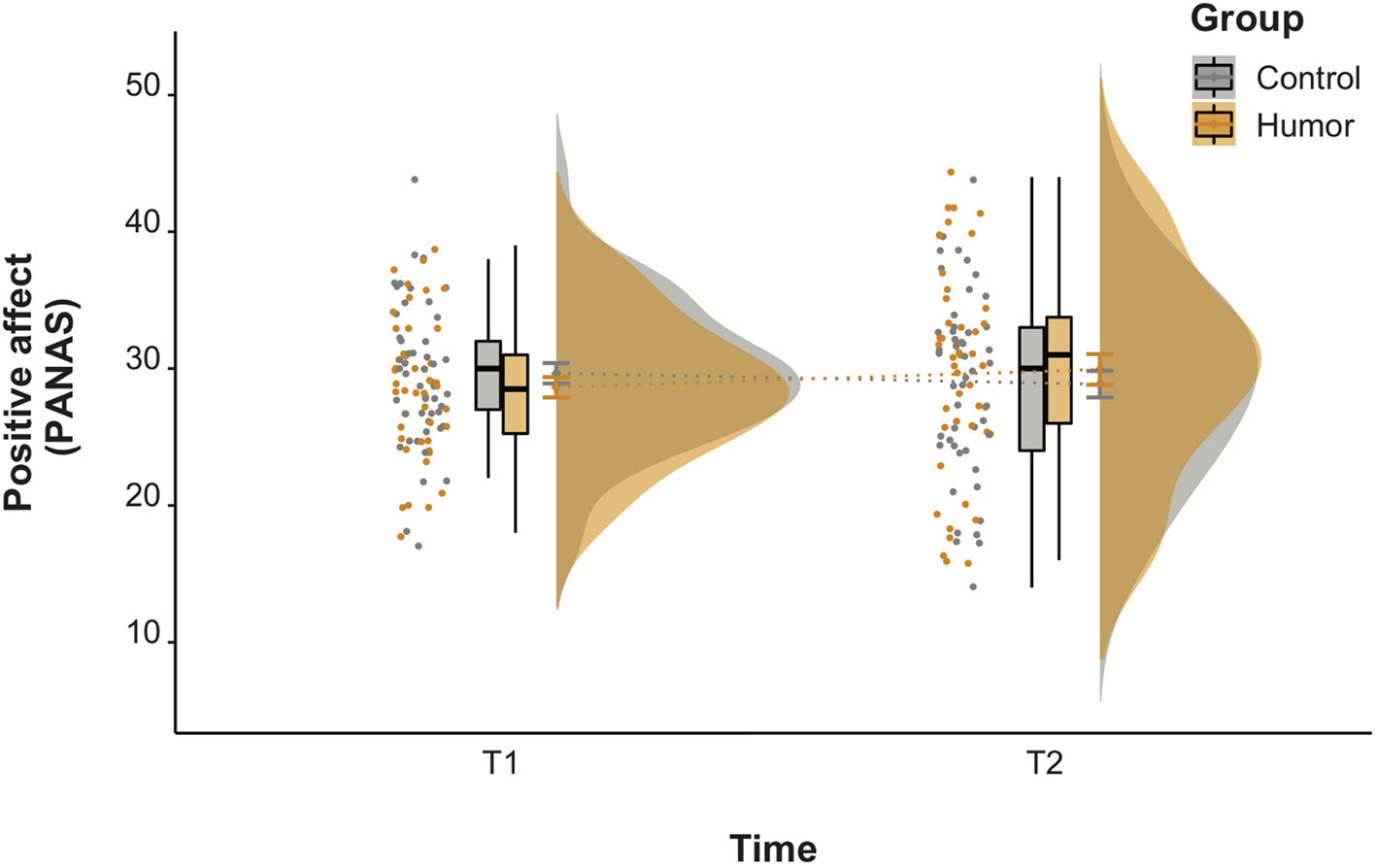
Positive affect before (T1) and after (T2) watching the neutral (control group) or funny (humor group) video clips.

In a second step, we tested whether the positive affect within the humor group improved significantly from before and after watching the video clips in comparison to the control group. We neither observed a significant main effect of group or time, but most importantly, the significant interaction of both factors, *F*_(1, 93)_ = 4.00, *p* < 0.05, η^2^ = 0.007. Further inspection showed a significant difference in mood change between the humor and the control group, *t*_(93)_ = 2.00, *p*<*0.0*5, *r* = 0.203, indicating an improvement in positive mood for the humor group, but not for the control group (Figure 3). We further investigated whether our mood manipulation may have also impacted the negative affect of both groups. The only significant effect we observed was a main effect of time, *F*_(1, 93)_ = 7.52, *p* < 0.01, η^2^ = 0.016. *Post Hoc* comparisons showed a decrease in negative affect over time, *t*_(93)_ = 2.74, *p* < 0.01, *r* = 0.274, however, the decrease did not differ between the two groups.

In a third step, we assessed valence and arousal levels as both factors have been suggested to affect emotional eating (Macht, 2008) as well as potential favorable effects of positive mood (Martin, 2002). As assumed, participants from the humor group rated their video clips as more arousing than the control group, *W* = 637, *p* < 0.001, *r* = −0.389 and rated themselves as being more aroused than the control group, *W* = 603, *p* < 0.001, *r* = −0.414. Moreover, the humor group reported higher self-valence levels than the control group, *W* = 909, *p* < 0.05, *r* = −0.198. There were no differences with respect to valence ratings of the video clips (see Table 4).

Taken together, our mood manipulation check indicates that the humor group improved in their positive affect after watching the video clips compared to the control group. This is backed by funniness and laughter ratings, which were also higher in the humor group in comparison to the control group. Additionally, participants of the humor group rated themselves as feeling more positive than the control group. Moreover, they indicated a higher level of self-arousal.

### Food Cue Attention Task

#### Response Times

To investigate the impact of humor on attention allocation toward food cues, a 2 × 2 × 2 (group × food cue × validity) linear mixed-effects model including the continuous predictor hunger ratings was calculated. It yielded a main effect of group, χ^2^(1) = 6.18, *p* < 0.05 and validity, χ^2^(1) = 3.87, *p* < 0.05, as well as the significant two-way interactions of group and hunger, χ^2^(1) = 7.54, *p* < 0.01. Moreover, we observed the significant three-way interaction of food cue, validity and hunger ratings, χ^2^(1) = 6.61, *p* < 0.05. Planned comparisons, which were directly encoded in the model, indicated faster response times for the humor compared to the control group, *b* = −22.0, *SE* = 8.86, *t*_(95.0)_ = 2.49, *p* < 0.05, while the comparison of valid and invalid trials was only marginally significant, *b* = −3.72, *SE* = 1.89, *t*_(94.3)_ = −1.97, *p* = 0.05.There was a tendency to respond faster to invalid trials.

**Figure 4:**
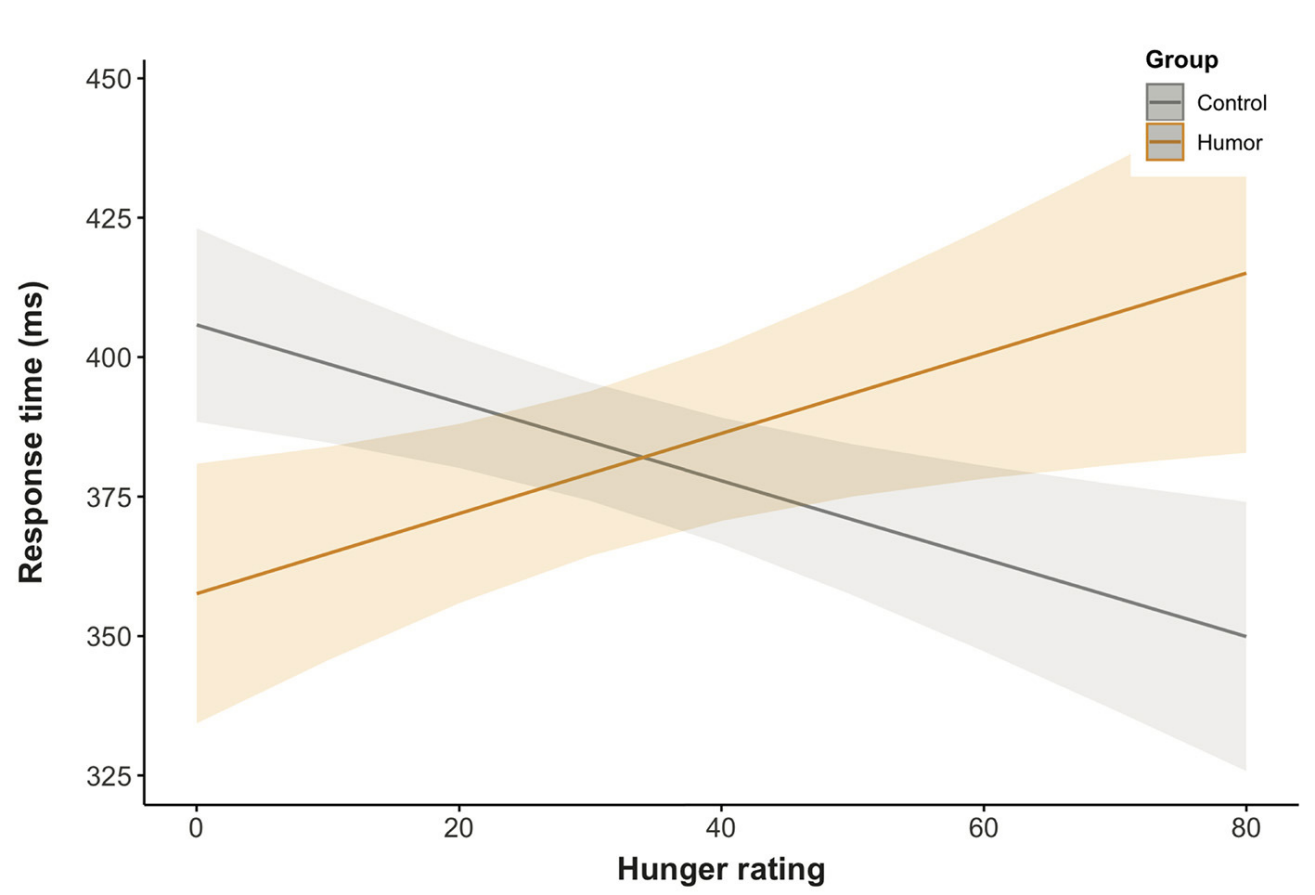
Predicted marginal effects of group and hunger ratings on response times.

Further inspection of the uncorrected contrasts for the interaction of group and hunger showed that response times of the control group decreased with increasing hunger, *t*_(99.2)_ = −1.99, *p* < 0.05, while there was the opposite trend for the humor group, *t*_(99.2)_ = 1.83, *p* = 0.07 (Figure 4). The difference between groups was found to be significant, *t*_(99.2)_ = −2.69, *p* < 0.01. After correcting for multiple testing, the group difference remained significant, *p* < 0.05, yet *p*-values of the group slopes increased to *p* = 0.10.

**Figure 5:**
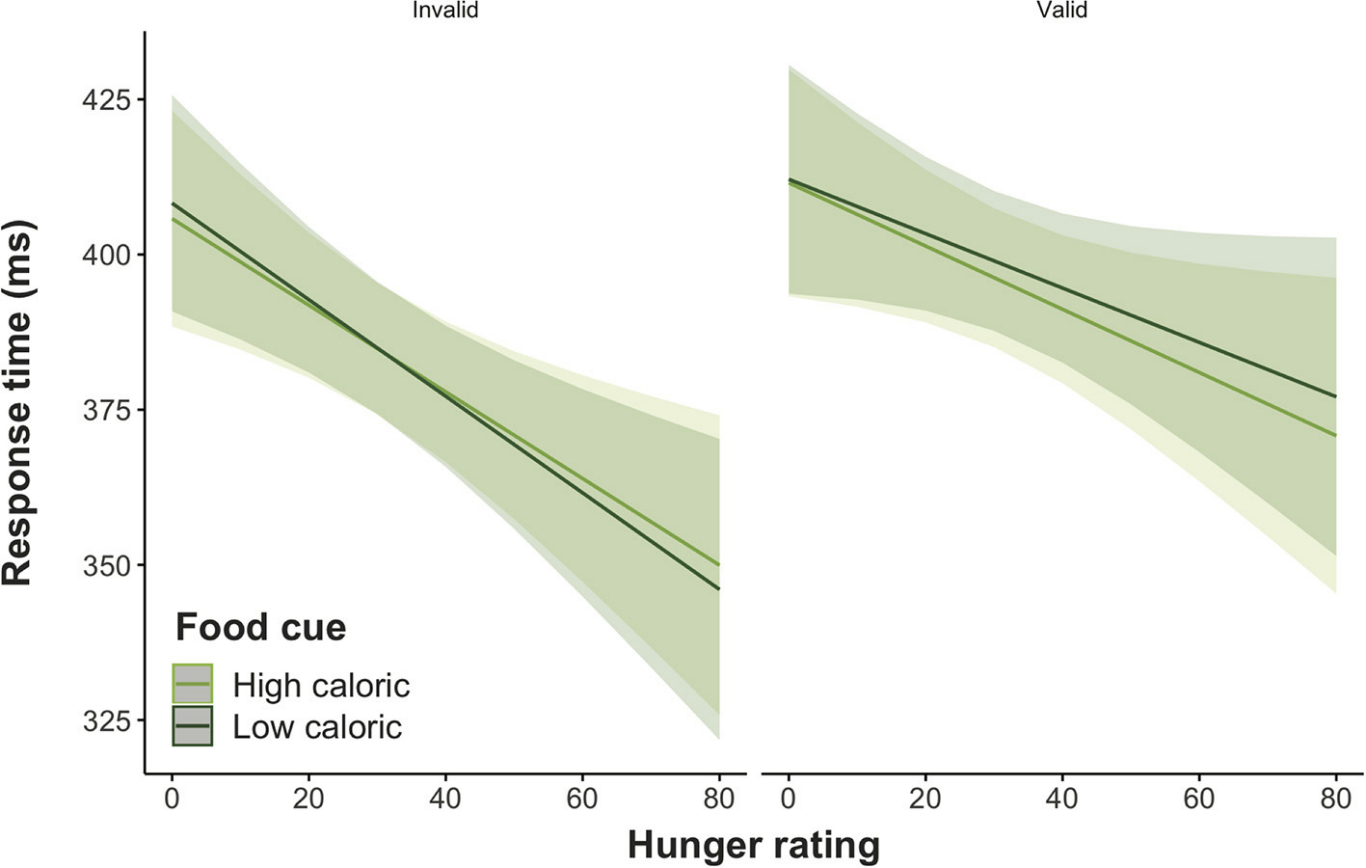
Predicted marginal effects of attentional engagement and disengagement on response times as a function of hunger ratings and food type.

Uncorrected, follow-up difference testing on the three-way interaction of food cue, validity and hunger ratings for attentional engagement (effect of food cues on valid trials) and disengagement (effect of food cues on invalid trials) indicated a significant difference between high and low caloric food cues for validly cued trials only, *t*_(14686)_ = −2.06, *p* < 0.05. As shown in Figure 5, response times in trials with high caloric food cues were faster compared to trials with low caloric food cues with the gap increasing with increasing hunger ratings. However, when correcting for multiple testing, the effect is no longer significant, *p* = 0.15.

#### Accuracy Data

The 2 × 2 × 2 (group × food cue × validity) × hunger ratings logistic mixed-effect analysis showed a main effect of validity, χ^2^(1) = 123.1, *p* < 0.001 with catch trials being less accurately responded to than invalid and valid trials, *b* = −3.59, *SE* = 0.43, *z* = −8.55, *p* < 0.001, however, there was no difference in accuracy rates between invalid and valid trials, p > 0.18.

In summary, analyses of the effects of humor on attentional bias toward food cues revealed a general tendency for the humor group to respond to food cues faster while satiated and slower when hungry, whereas the opposite pattern was found for the control group. Additionally, there is an indication that attentional engagement may differ between healthy and unhealthy food items when the homeostatic state is taken into account. Results point toward a preference for attending to unhealthy food over healthy food. Findings indicate that participants are faster in disengaging from healthy food than attending to it. The interaction term may be driven by the differences in attentional engagement/disengagement and healthy and unhealthy food preferences, which become more pronounced with increasing hunger.

## Discussion

The present study set out to investigate the impact of humor on attention allocation and reorientation toward food cues and its effect on healthy and unhealthy food preferences. For this purpose, we successfully induced positive affect in a group of healthy-weight individuals and compared their performance in the FCAT to a neutral control group. The FCAT is a modified Posner cueing task, which enabled us to assess attentional engagement and disengagement biases toward high and low caloric foods. Hunger ratings were included in the analysis, as they have been found to significantly improve model fit. Our results were as follows: (a) humor showed an impact on the response toward food cues which was modulated by hunger; (b) with increasing hunger, there was a trend for an increasing difference in engagement bias between healthy and unhealthy food preferences, (c) there were no differences in engagement and disengagement biases in healthy and unhealthy food preferences as a function of humor, and (d) overall emotional eating differed in the humor group from that in the control group, particularly for anxiety.

### Humor and Hunger Affect the Response Toward Food Cues

In line with our expectations, we observed an impact of humor on the response toward food cues, which was modulated by hunger ratings. There was a significant difference between the humor and the control group, with the humor group being faster than the control group to attend to food images when satiated and the control group being faster than the humor group when hungry.

Hunger seems to play a decisive role, when assessing the impact of humor on the attentional bias toward food cues (e.g., Castellanos et al., 2009; Tapper et al., 2010; Jonker et al., 2020). Hunger indicates the balance (satiation) or imbalance (hunger) of the individual's homeostatic state. We found the humor group to respond faster to food cues compared to the control group when homeostasis was in balance. As we presented food cues peripherally, these findings are consistent with the assumptions of the Broaden-and-Build Theory and earlier models (Easterbrook, 1959; Fredrickson, 1998, 2001), that postulate an increase in responsiveness to peripheral cues through a broadening of attentional breadth due to positive emotions. However, in case of an imbalance, we observed a response time advantage for the control group compared to the humor group. In healthy-weight individuals (which the participants of the present study were) food deprivation has been shown to increase the intrinsic reward value attributed to food, thus making it more likely to locate attention toward these items as well as to initiate approach behavior (Higgs et al., 2017; Jonker et al., 2020). As the value attached to food decreases with increasing satiation, previous studies reported an attentional bias for individuals who had fasted only, not for satiated ones (e.g., Castellanos et al., 2009). Comparing the control group to the humor group, this resembles the response pattern we observed for these individuals in the present study. There seems to be some mechanism, triggered by positive affect, that prevents the humor group from rapidly responding food cues, thus from displaying the commonly observed approach behavior toward food cues for fasted healthy-weight individuals (e.g., Channon and Hayward, 1990; Mogg et al., 1998; Castellanos et al., 2009; Jonker et al., 2020).

One possible explanation has been put forward by Adam and Epel (2007), and compliments findings from our group (Froehlich et al., 2021). Acute (aversive) stress, which is heavily intertwined with emotions (Lazarus, 2006; Evers et al., 2018), potentially increases cortisol levels and activates the reward system, making food cues intrinsically more valuable (Adam and Epel, 2007). In fact, the only study investigating (negative) affect and attentional bias toward food cues seems to support this assumption. Negative mood was found to increase attentional bias toward food cues (Hepworth et al., 2010). The present study did not induce negative mood or affect (nor did we measure stress). However, Froehlich et al. (2021) were able to show that a successful induction of positive affect by means of humorous video clips (the same clips as here) effectively decreases cortisol levels compared to a neutral control group. Thus, the mechanism inhibiting fast responses toward food cues in the humor group in the present study might be because of lower cortisol levels, a lower activation of the reward system and thus, a lower incentive value of food cues than in the control group. Even though the proposed mechanism is highly speculative and needs further investigation, a humorous intervention may possibly help to prevent overeating when hungry.

Yet, this conclusion seems to stand in stark contrast with findings indicating a higher risk behavior including alcohol consumption, drug intake, and binge eating in individuals experiencing intense positive emotions (Martin, 2002; Cyders and Smith, 2008; Evers et al., 2013) as well as an increased food intake when feeling positive affect (Evers et al., 2013, 2018; Cardi et al., 2015). However, the present study aimed at assessing the impact of humor on attentional bias toward food, rather than the amount of food intake. We would highly recommend to investigate how actual eating behavior and attentional response toward food cues relate to each other as a function of humor/positive affect. Additionally, there is evidence that stress and negative mood appear to be strong predictors for unhealthy eating behaviors, such as binge eating as well (Wolff et al., 2000; Danner et al., 2013). Similar observations, including comparable increased food intake under negative and positive affect compared to neutral, have led researchers to discuss whether valence (positive vs. negative) or the level of arousal (high vs. low) of the emotions may be the primary contributing factor underlying reported findings (e.g., Cools et al., 1992; Evers et al., 2013, 2018). Further, independently from differentiating between the valence and arousal of emotions, the level of arousal itself provides ample room of research. On the one hand, it has been shown that low levels of arousal due to positive affect increase cognitive self-control, whereas the opposite is true for high levels of arousal (Fedorikhin and Patrick, 2010). On the other hand, higher levels of arousal are thought to increase activation of the autonomic nervous system, thus leading to physiological changes that in turn increase satiety (Evers et al., 2018). In fact, the latter is what we observed in the present study. The humor group reported higher levels of self-arousal than the control group and showed a diminished response toward food cues when compared to the control group; this could reflect a satiating influence of the mood inducement manipulation. A final conclusion regarding the underlying mechanisms of our findings remains open, as we were not able to obtain cortisol levels of the participants due to the study being conducted online as well as the fact that incorporating arousal levels in the statistical model did not improve model fit.

### Hunger and Attentional Engagement/Disengagement Toward Healthy and Unhealthy Food Items

While previous studies agree on the fact that attentional bias toward food cues is likely driven by states of fasting and satiety, they provide conflicting results regarding the impact of hunger on attentional engagement and disengagement. For instance, findings reported by Jonker et al. (2020) point toward an exclusive influence of hunger on attentional engagement without affecting disengagement from food cues. In contrast, Tapper et al. (2010) observed only effects of attentional disengagement in hungry individuals, but none for attentional engagement to food cues. There are two possible reasons for these seemingly inconsistent findings. First, while Jonker et al. (2020) used two groups of fasted and satiated individuals, Tapper et al. (2010) operationalized hunger as a continuous variable *via* a VAS, similarly to the present study. Second, the food stimuli consisted of either sweet and savory high caloric food items (Jonker et al., 2020) or appetizing and bland (e.g., lettuce, rice cakes) food images (Tapper et al., 2010). In fact, the stimuli used in the present study captured all of these characteristics but were additionally matched for caloric content and thus possibly account for the previously conflicting findings. We observed a trend toward an increasing difference in engagement bias to healthy and unhealthy food items indicating an engagement bias toward unhealthy food which seems to be more pronounced with increasing hunger. This result indicates that in a state of hunger, attention is more likely to be allocated toward unhealthy food items than to healthy ones. This is in line with previous findings, that showed an attentional bias toward high caloric food words for fasted individuals but no attentional bias toward low caloric food words (Placanica et al., 2002).

From an evolutionary point of view, it seems highly sensible, when in a heightened state of homeostatic imbalance and in dire need of energy uptake, to allocate attention toward food that delivers this energy quickly and efficiently. Thus, unhealthy but energy-rich food becomes more attractive, is attended to faster and captures attention more effectively than healthy but energy-low food. This exact same mechanism may also explain why dietary success can be compromised when periods of fasting are too long. Not only is hunger known to impair cognitive self-control (e.g., Loeber et al., 2013), it may also drive one to preferably attend unhealthy food items and ignore healthy ones. However, this is highly speculative as our results failed to reach significance after correcting for multiple testing. Therefore, there is a need to replicate the present findings.

### Humor and Attentional Bias Toward Food Cues

Despite successfully inducing positive affect, we did not observe a differential impact of humor on attentional engagement and disengagement (i.e., on the attentional bias toward food cues) for healthy and unhealthy food. Several reasons may account for this. First, faster response times to peripherally presented food cues of satiated individuals in the humor group point toward a broadening of attentional breadth compared to the control group. This is consistent with the observation by Wadlinger and Isaacowitz (2006) that positive mood increases the frequency of attending to peripheral cues, however, the cues have to be highly-valenced positive stimuli. Individuals of both groups in the present study scored roughly within the normal range of emotional eating, which could indicate that food stimuli elicited just enough internal valence to draw attention, but not enough to trigger group differences in attentional engagement and disengagement. Second, some studies observed distinctive differences in attentional engagement and disengagement at only very short stimulus presentation times (100 ms; Nijs et al., 2010; Jonker et al., 2019, 2020) thus at markedly shorter presentation duration than used in the present study (i.e., 500 ms). In fact, presentation times of 500 ms lie within the dwell time of attention (Bundesen and Habekost, 2008) and have successfully been used to observe attentional bias toward food cues (e.g., Mogg et al., 1998; Hepworth et al., 2010; Tapper et al., 2010). It seems, however, that shorter stimulus presentation times might be beneficial in order to be able to differentiate between engagement and disengagement. On top of that, the relatively long presentation times of the food cues may have caused participants to voluntarily shift attention to prepare for the upcoming target presentation (e.g., Born et al., 2011). However, if this were the case, then there should have been no (marginally significant) main effect of validity. Further research is needed to clearly discern between voluntary maintenance of attention and (true) disengagement processes. Third, due to concerns of study length and participants' compliance, we did not include neutral stimuli in the FCAT as previous studies did. Forth, we used response times to assess attentional bias toward food cues, but other studies also used eye tracking measures, which do not necessarily produce results equivalent to response times (e.g., Castellanos et al., 2009). Finally, the findings by Gardner et al. (2014) may explain why the present study did not observe a difference in preferences for high and low caloric food as a function of humor. A positive mood increases the saliency of individual long-term goals, such as healthy living, which in turn leads to a preference for healthy food over indulgent food. However, if positive affect is threatened, individuals are less willing to try healthy food (Andrade, 2005). In a similar manner, Tapper et al. (2010) stress the importance of goal-relevance of the stimuli for potentially affecting attentional bias. It was beyond the scope of the present study to assess short- and long-term goals, as well as whether individuals felt mood lifting or threatening effects.

### Conclusion and Future Research

The present study was the first to investigate the impact of humor on healthy eating behavior by investigating its influence on attentional bias toward food cues as well as on healthy and unhealthy food preferences. Additionally, contrary to previous studies, we made use of all individual data points by applying linear mixed models to the data, instead of calculating difference scores or ANOVAs. The results point toward a pivotal role of hunger when assessing responses toward food cues. Most importantly, humor seems to counteract aversive consequences of hunger on attention allocation, possibly by decreasing cortisol levels, thus reducing the activation of the reward system and therefore lowering the incentive value attached to food. However, the proposed underlying mechanisms need to be validated by future research. Moreover, hunger seems to shift attention away from healthy food items toward unhealthy food. In a similar manner, we call for a replication of our results given the space amount of empirical data. The present study was conducted as an online study and therefore lacked the highly controlled setting of a laboratory experiment. Even though results from online studies seem to be comparable to lab-based studies (Nussenbaum et al., 2020), there is ample room for susceptibility. Combining experimental settings with more naturalistic settings, such as self-reported daily-life eating behaviors, would not only improve internal validity but also external validity (cf. Reichenberger et al., 2020). Moreover, employing very short and intermediate stimulus durations may positively affect the examination of attentional engagement and disengagement biases. Also, using neutral stimuli images as well as varying the degree of valence of the images may impact food preferences and attentional bias. Furthermore, future studies should investigate whether it makes a difference if hours of fasting or hunger levels are used to assess the state of satiety. With increasing popularity of fasting diets and individuals being used to fasting for longer periods, time of fasting may not be as conclusive as hunger ratings. Additionally, research should look at the actual link between positive and negative affect, attentional bias and concrete food intake considering not only spatial attentional bias but temporal attentional bias (e.g., Neimeijer et al., 2017) as well. Besides the valence of emotions, arousal seems to play a prominent role in affecting attention and eating behavior; this calls for more research focusing on this distinctive contribution to previous findings. Extending experimental methods such as utilizing eye-tracking, neuroimaging or neuro endocrinological data may help. Lastly, it is still an open issue as to how exactly (positive) emotions affect food consumption, not only in healthy-weight individuals but also in restrained or obese eaters, or individuals with eating disorders.

## Data Availability Statement

The raw data supporting the conclusions of this article will be made available by the authors, without undue reservation.

## Ethics Statement

The studies involving human participants were reviewed and approved by Ethics Committee of the Humboldt Universität zu Berlin. The patients/participants provided their written informed consent to participate in this study.

## Author Contributions

EF, SP, and RM contributed to the conception of the study. LS and EF programmed the task, ran, monitored the experiment, performed statistical analyses, and wrote the first draft of the manuscript. EF prepared the figures. All authors contributed to the revised version of the manuscript.

## Funding

The present study was funded by grants from the German Ministry of Education and Research (BMBF) and the State of Brandenburg (DZD, FKZ 82DZD03D03).

## Conflict of Interest

The authors declare that the research was conducted in the absence of any commercial or financial relationships that could be construed as a potential conflict of interest.

## Publisher's Note

All claims expressed in this article are solely those of the authors and do not necessarily represent those of their affiliated organizations, or those of the publisher, the editors and the reviewers. Any product that may be evaluated in this article, or claim that may be made by its manufacturer, is not guaranteed or endorsed by the publisher.
