# Supplementary material for: Humor and Hunger Affect the Response Toward Food Cues
Source: Front Psychol. 2021 Sep 16;12:680508. doi: 10.3389/fpsyg.2021.680508 (PMC8481376; doi:10.3389/fpsyg.2021.680508)
Supplement: Supplementary file 1 [file Data_Sheet_1.docx]

Supplementary Material

# Supplementary Tables

Table S1. Item numbers and descriptions of high and low caloric food cues

| High caloric food items | | Low caloric food items | |
| --- | --- | --- | --- |
| Number | Item description | Number | Item Description |
| 10 | Spaghetti with tomato sauce | 141 | Lasagna |
| 58 | Ham Sandwich | 195 | Cucumber slice |
| 86 | Cheese burger, French fries | 204 | Apples |
| 102 | Hard candies | 221 | Oranges |
| 117 | Chips | 243 | Strawberries |
| 148 | Round shortbread cookies | 249 | Cauliflower |
| 162 | Chocolate cream cake | 254 | Figs |
| 173 | Chocolate bar filled with milk cream | 256 | Grapefruit |
| 180 | Muesli bar (oatmeal) | 257 | Lettuce (lollo rosso) |
| 187 | Croissant | 270 | Corn (on a cob) |
| 236 | Bowl of rice | 271 | Parsley |
| 279 | Sushi | 316 | Spinach-potato casserole |
| 308 | Salmon sushi | 334 | Carrots |
| 321 | Egg boiled | 337 | Meat fillets and vegetables, grilled |
| 338 | Mixed licorice | 369 | Sushi roll with cucumber |
| 344 | Brownie with nuts | 389 | Cantaloup |
| 348 | Rusk | 428 | Artichoke |
| 373 | Vanilla ice cream | 434 | Leek |
| 374 | Donut with icing | 437 | Potatoes |
| 423 | Bread roll | 438 | Onion |
| 425 | Bread roll | 447 | Mixed vegetables |
| 439 | Toast / white bread | 449 | Garden radish |
| 451 | Hazelnut | 460 | Tomato |
| 475 | Apple danish | 477 | Glass of gherkins |
| 481 | Flat cake with salad | 478 | Pineapple |
| 494 | Pretzel | 528 | Peaches |
| 498 | Meat pie | 529 | Mixed vegetables |
| 505 | Cookies | 530 | Strawberry, half |
| 516 | Edam cheese | 521 | Raspberries |
| 519 | Camembert | 540 | Beefsteak, raw |
| 525 | Walnut | 541 | Chicken, raw |
| 577 | Lasagna | 548 | Cooked ham |
| 602 | Cornflakes with milk | 558 | Asparagus with bechamel sauce |
| 619 | Ramen noodles | 561 | Cutlet |
| 635 | Cookie | 601 | Tomato soup |
| 682 | Sandwich with cheese | 654 | Rice |
| 712 | Tortellini | 693 | Pumpkin soup |
| 729 | Ice cream sundae | 743 | Artichoke |
| 802 | Brownie | 746 | Lime |
| 812 | Wheat bun | 750 | Bananas |
| 816 | Chocolate muffin | 772 | Cherries |
| 839 | Wheat bread | 788 | Celery |
| 857 | Garlic | 793 | Broccoli |
| 859 | Chocolate cereal | 862 | Corn |

*Note.* Food items were taken from the Salzburg Food Pics database (Blechert et al., 2014; 2019).

Table S2. Item characteristics for high and low caloric food cues

|  | High caloric food items | | Low caloric food items | |  |
| --- | --- | --- | --- | --- | --- |
|  | *M* (*SD*) | range | *M* (*SD*) | range | *p* |
| kcal_100g | 320.29 (20.96) | 139–654 | 55.64 (5.51) | 9–123 | < .001 |
|  |  |  |  |  |  |
| *Physical properties* | | | | | |
| red | .47 (.01) | .34–.59 | .46 (.02) | .28–.75 | > .78 |
| green | .33 (.004) | .27–.37 | .34 (.01) | .12–.49 | > .28 |
| blue | .20 (.01) | .10–.30 | .20 (.01) | .04–.37 | > .49 |
| size | .29 (.02) | .04–.50 | .32 (.02) | .08–.62 | > .30 |
| intensity | 30.29 (2.48) | 5.06–82.10 | 34.66 (2.55) | 8.44–77.98 | > .22 |
| contrast | 52.23 (2.31) | 24.80–89.43 | 50.74 (1.97) | 20.19–86.24 | > .63 |
| complexity | .09 (.01) | .02–.15 | .10 (.01) | .02–.17 | > .17 |
|  |  |  |  |  |  |
| *Food related properties* | | | | | |
| kcal_total | 241.28 (26.53) | 36.50–652.08 | 193.75 (28.37) | 0.99–702.00 | > .22 |
| valence^a^ | 53.38 (1.23) | 31.1–70.9 | 55.89 (1.35) | 36.20–75.60 | > .17 |
| valence^b^ | 52.32 (1.46) | 32.5–72.8 | 53.3 (1.71) | 14.4–76.2 | > .66 |
| arousal^a^ | 32.31 (1.32) | 14.4–53.3 | 30.59 (1.34) | 17.0–51.0 | > .36 |
| arousal^b^ | 31.00 (1.24) | 12.7–46.3 | 31.26 (1.88) | 3.7–55.7 | > .91 |
| craving^a^ | 29.95 (1.25) | 8.8–47.1 | 30.98 (2.02) | 7.2–66.4 | > .67 |
| craving^b^ | 33.76 (1.67) | 11.2–57.6 | 32.90 (2.49) | 2.2–69.2 | > .77 |
| familiarity^a^ | 94.38 (1.30) | 66.7–100.0 | 97.12 (0.86) | 76.5–100.9 | > .08 |
| familiarity^b^ | 94.47 (1.24) | 66.7–100.0 | 92.08 (1.82) | 40.0–100.0 | > .28 |

*Note. M* = mean. *SD* = standard deviation

^a^ratings omnivores (females)

^b^ratings omnivores (males)

**References**

Blechert, J., Lender, A., Polk, S., Busch, N. A., & Ohla, K. (2019). Food-Pics_Extended—An Image Database for Experimental Research on Eating and Appetite: Additional Images, Normative Ratings and an Updated Review. *Frontiers in Psychology*, *10*, 307. doi.org/10.3389/fpsyg.2019.00307

Blechert, J., Meule, A., Busch, N. A., & Ohla, K. (2014). Food-pics: An image database for experimental research on eating and appetite. *Frontiers in Psychology*, *5,* 617. doi.org/10.3389/fpsyg.2014.00617
